# Fatal prostate cancer incidence trends in the United States and England by race, stage, and treatment

**DOI:** 10.1038/s41416-020-0859-x

**Published:** 2020-05-20

**Authors:** Eboneé N. Butler, Scott P. Kelly, Victoria H. Coupland, Philip S. Rosenberg, Michael B. Cook

**Affiliations:** 1 Division of Cancer Epidemiology and Genetics, National Cancer Institute, 20892 Bethesda, MD USA; 2 National Cancer Registration and Analysis Service, Public Health England, Wellington House, SE1 8UG London, UK

**Keywords:** Epidemiology, Population screening

## Abstract

**Background:**

Differential uptake of prostate-specific antigen testing in the US and UK has been linked to between-country differences for prostate cancer incidence. We examined stage-specific *fatal* prostate cancer incidence trends in the US and England, by treatment and race/ethnicity.

**Methods:**

Using data from the National Cancer Institute’s Surveillance, Epidemiology, and End Results program and Public Health England’s National Cancer Registration and Analysis Service, we identified prostate cancer patients diagnosed between 1995 and 2005, aged 45–84 years. Fatal prostate cancer was defined as death attributed to the disease within 10 years of diagnosis. We used age–period–cohort models to assess trends in fatal prostate cancer incidence.

**Results:**

Fatal prostate cancer incidence declined in the US by −7.5% each year and increased in England by 7.7% annually. These trends were primarily driven by locoregional disease in the US and distant disease in England. Black men in both countries had twofold to threefold higher fatal prostate cancer incidence rates, when compared with their white counterparts; however, receipt of radical prostatectomy lessened this disparity.

**Conclusions:**

We report a significant increasing rate of fatal prostate cancer incidence among English men. The black–white racial disparity appears pervasive but is attenuated among those who received radical prostatectomy in the US.

## Background

Following the introduction of prostate-specific antigen (PSA) testing in the United States (US), fatal prostate cancer incidence declined between 1995 and 2002 at an average rate of 5% per year.^1^ Defined as death from the disease within 10 years of diagnosis, ‘fatal prostate cancer’ represents a non-indolent subset of the disease that is less sensitive to lead time bias in descriptive epidemiology studies^2^ and allows investigators to better track incidence trends alongside contemporary clinical practices. Indeed, fatal prostate cancer incidence in the US declined by 50% between 1972 and 2002, which coincided with uptake of radical prostatectomy (RP) and improvements in early disease detection.^3–5^ By contrast, the United Kingdom (UK) has less readily adopted radical surgical treatments and population screening during this period, with a concomitant gradual increase in overall incidence (though less is known regarding fatal prostate cancer incidence, specifically).^6^ A comparison of fatal prostate cancer incidence trends in the US and UK—two countries that have vastly different PSA testing histories—may help further illuminate the relationship between population-based screening and detection of clinically relevant disease.

Despite evidence from observational studies suggesting a possible link between PSA testing and reduced prostate-cancer mortality, randomised controlled trials in the US and Europe have failed to demonstrate a positive net benefit for the screening modality.^7–9^ Rather, serum PSA testing has highlighted the challenge of detecting non-indolent forms of prostate cancer.^10^ As the US embraces calls for fewer tests due to concerns of overtreatment and overdiagnosis,^11,12^ the PSA screening rate in the UK remains relatively low, with a modest increase observed from 2% at the turn of the century to a present estimated annual uptake of 6% for UK men aged ≥45 years.^13–15^ By comparison, PSA test use among US men aged ≥50 years were approximately 40% in the year 2000 with a subsequent decline to 37% in 2015.^16^ Current projections for the UK indicate a continued increase in prostate cancer incidence through the year 2035.^17^ What remains to be known is the extent to which UK incidence rates reflect trends for fatal prostate cancer. Further, the persistent disparity in prostate cancer incidence and mortality among men of African ancestry in the US and UK warrants special attention, given the occurrence of poor outcomes for these men across the two varying geographical and cultural contexts.^18–20^

In this study, we assessed fatal prostate cancer incidence through comparisons of geography (United States vs. England), race (black vs. white), and initial treatment (RP) for the period 1995–2005; the selected time period follows the introduction of PSA screening and the depletion of a prevalent disease reservoir in the US, thus allowing us to examine trends that are unobscured by large fluctuations in PSA testing. We emphasised RP specifically as its uptake as a definitive treatment for prostate cancer roughly coincides with the initial period of widespread PSA testing in the US. Finally, we selected England for comparison to the US, as it is the most populous and ethnically diverse country within the UK.

## Methods

### Data sources

#### National Cancer Registries

The National Cancer Institute’s Surveillance, Epidemiology, and End Results program consists of 18 population-based registries (SEER 18) that capture 28% of all cancers diagnosed in the United States (US)^21^ (November 2018 Submission). Using SEER 18, we identified US men who received a first primary diagnosis of prostate cancer between 1995 and 2015 (*International Classification of Diseases for Oncology*, Third edition, code C619). We restricted our study population to those who were aged 45–84 years at the time of diagnosis and excluded cases who were diagnosed by death certificate only. We defined fatal prostate cancer as death attributed to the disease within 10 years of diagnosis, in line with prior work demonstrating that 70% of prostate cancer-specific deaths in SEER were captured within a 10-year window.^21^ The SEER cause-specific death algorithm accounts for tumour sequence and thus is designed to attribute cause of death to the primary cancer site when applicable. In total, we described overall incidence for 884,496 US men diagnosed between 1995 and 2015 to aid the interpretation of our fatal prostate cancer incidence projections; a subtotal of 395,209 men diagnosed between 1995 and 2005 were eligible for our primary analysis of fatal prostate cancer incidence. For each US prostate cancer case, we extracted data on SEER historic stage (i.e. locoregional or distant), grade, and type of surgery received during first course of treatment (i.e. RP, other surgery, none).

The National Cancer Registration and Analysis Service (NCRAS) collects data on all cancers diagnosed in England.^22^ To define our English study population, we applied inclusion and exclusion criteria equivalent to that used to define the US study population. Namely, we identified men who received a first primary diagnosis of prostate cancer between 1995 and 2015, aged 45–84 years at the time of diagnosis. We excluded prostate cancer registrations that only had information from a death certificate. In total, we described overall incidence for 506,736 English men diagnosed between 1995 and 2015 in order to aid the interpretation of our fatal prostate cancer incidence projections; a subtotal of 228,615 men diagnosed between 1995 and 2005 were eligible for our primary analysis of fatal prostate cancer incidence. Similar to SEER, the NCRAS uses an algorithm that incorporates tumour sequence to determine the underlying cause of death. For each patient in the English population, we extracted data on tumour–node–metastasis (TNM) stage, grade, and type of surgery received during first course of treatment (i.e. RP, other treatment, none). English patients with a TNM stage of I, II, or III were classified as having ‘locoregional’ disease; patients with TNM stage IV tumours were classified as having ‘distant’ disease. The NCRAS uses the internationally recognised TNM classification schema, maintained by the Union for International Cancer Control. When used as a dichotomous variable (i.e. locoregional vs. distant), distant stage diagnoses refer to cancers with metastases. This binary classification schema is comparable to the SEER historic stage variable.

#### Census data

To calculate age-standardised and age-specific incidence rates in the US, we obtained single-age census population estimates through SEER 18 for each calendar year between 1995 and 2015. We also obtained race-specific population estimates to enable estimation of race-specific incidence rates. Race categories were based on US census designations and men were categorised as white, black, American Indian/Alaskan Native, Asian or Pacific Islander, or unknown.

Similarly, to estimate age-standardised and age-specific incidence rates in the English population, we obtained single-age population estimates for each calendar year in our study through the Office for National Statistics (1995–2014). The Office for National Statistics provides race/ethnicity-specific population estimates for 5-year age groups in each official census year. Thus we used spline interpolation to estimate age- and race/ethnicity-specific population counts for the intercensal years between 1991, 2001, and 2011. White men included those with self-reported white race and self-reported ethnicity as British, Irish, other, or undefined. Black men included those with self-reported black race and self-reported ethnicity of Caribbean, African, or undefined. Men who reported race other than black or white were classified as ‘other’. For both countries, we limited our race/ethnicity-specific analyses to white and black men.

### Data analysis

#### Missing data

The US data set had missing data for stage (4.1%), race (2.3%), grade (5.1%), and surgery (1.0%). The English data set had missing data for race (20.1%), grade (40.0%), and stage (72.8%). To approximate plausible values for missing data, we performed multiple imputations by chained equations using the R package MICE^23^; values coded as ‘unknown’ were assigned as missing prior to imputation. Our prediction models included data on age, year of diagnosis, survival months, stage, grade, treatment type (RP, other treatment, none), and cause of death. We included ‘grade’ for imputation only, which was coded from I to IV in the US data set. In the English data set, grade was recorded for two sections of the tumour with scores ranging from 1 to 5 for degree of severity; both grades were combined to create a composite score ranging from 2 to 10. Data were imputed under a missing at random assumption. We imputed 10 data sets using 15 iterations per cycle and subsequently pooled data using Rubin’s rule to examine the beta coefficients for the association between prostate cancer-specific death and imputed variables^24^; pooled estimates reflect a relative efficiency of 96% in the US data set and 93% in the English data set. We used the first imputed data set generated for each country for our primary analysis of fatal prostate cancer incidence trends by race, stage, and treatment, as beta estimates for the imputed variables were comparable across the 10 imputations (Supplementary Tables 1 and 2).

#### Incidence trends and age–period–cohort models

For each country, we calculated age-standardised rates using the World Standard Million population and age-specific incidence rates overall and by race.^25^ All incidence rates are reported in the units of per 100,000 person (male)-years. We describe trends as the estimated annual percentage change (EAPC) across the specified time period and used joinpoint regression modelling to identify calendar periods with a change in the rate of disease occurrence;^26^ significant joinpoints were identified through a series of permutation tests with Bonferroni-corrected *p* values.

We fit age–period–cohort models to estimate the EAPC for fatal prostate cancer incidence rates, both overall and by stage of disease (locoregional/distant);^27,28^ observed trends are described for the period between 1995 and 2005. For each age-period cohort model, we examined the normal probability plot of residuals as a measure for goodness of fit; tests of normality were evaluated using Anderson–Darling and Lilliefors criteria. We compared trends for incidence rates between the US and England using Wald chi-square tests with a significance level of 0.05.^29^ Within country, we estimated rate ratios for black-to-white comparisons of fitted temporal trends, which model incidence rates for each period adjusted for cohort deviations and age. In addition, we used age–period–cohort forecast models to describe projected trends for fatal prostate cancer incidence between 2006 and 2015.^30^ We performed statistical analyses in Matlab version 2018b (Mathworks Inc., Natick, MA, USA) and R version 3.5.0 (R Foundation for Statistical Computing, Vienna, Austria).

## Results

### Incidence of fatal prostate cancer in the US and England

**Fig. 1:**
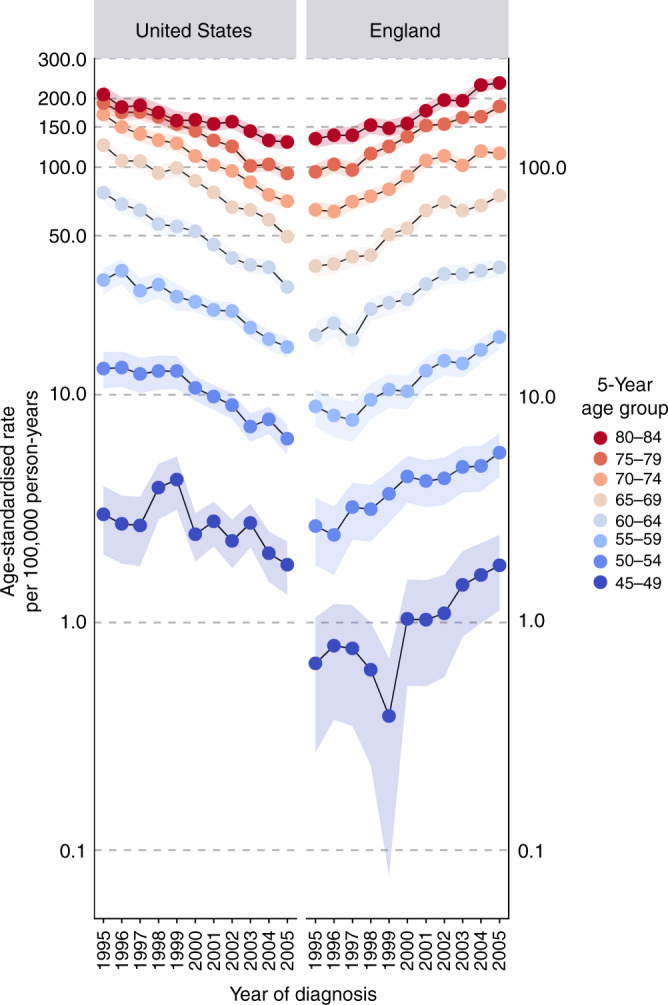
Age-specific fatal prostate cancer incidence rates by country and year of diagnosis, 1995–2005. Incidence rates are described for 5-year age groups (range 45–84 years) and plotted on the logarithmic scale.

The normal probability plot of residuals for each country-specific age–period–cohort model demonstrated overall good fit; thus all models were suitable for between-country comparisons. Between 1995 and 2005, 11% of US men newly diagnosed with prostate cancer died from their disease within 10 years (*n* = 43,020), compared with 17% of men diagnosed in England (*n* = 39,249). In 1995, the US outpaced England 3-to-1 with an age-standardised fatal prostate cancer incidence rate of 85.8 per 100,000 (Table 1). By 2005, the incidence rates of fatal prostate cancer between the two countries had switched owing to a 55% decline in the US (from 85.8 to 38.3 per 100,000) and a corresponding 100% increase in England (from 24.8 to 49.2 per 100,000). In the US, the fatal prostate cancer incidence rate declined for each single age group (Wald test: *Χ*^2^ = 115.0, df = 40, *p* < 0.001), with the sharpest declines occurring among those aged 60–69 years (Fig. 1). By contrast, the EAPC for fatal prostate cancer incidence in England increased at every age (Wald test: *Χ*^2^ = 62.2, df = 40, *p* = 0.01), with the highest rate of increase observed among men aged 45–49 years.

**Table 1:** Fatal prostate cancer incidence rates in the United States (US) and England between 1995 and 2005, among men aged 45–84 years.

	US	England	US: England incidence rate ratio
	EAPC: −7.5%	EAPC: 7.7%	
Year of diagnosis	Incidence rate^a^ per 100,000	Incidence rate^a^ per 100,000	OR (95% CI)
1995	85.8	24.8	3.5 (3.1–3.9)
1996	76.7	26.0	2.9 (2.6–3.3)
1997	73.2	26.6	2.8 (2.5–3.1)
1998	68.5	29.8	2.3 (2.1–2.6)
1999	66.2	32.3	2.0 (1.8–2.3)
2000	59.8	35.1	1.7 (1.5–1.9)
2001	54.4	40.9	1.3 (1.2–1.5)
2002	50.9	43.7	1.2 (1.0–1.3)
2003	45.5	43.0	1.1 (0.9–1.2)
2004	42.5	46.5	0.9 (0.8–1.0)
2005	38.3	49.2	0.8 (0.7–0.9)

*EAPC* estimated annual percentage change.

^a^Adjusted for cohort deviations and age at diagnosis.

**Fig. 2:**
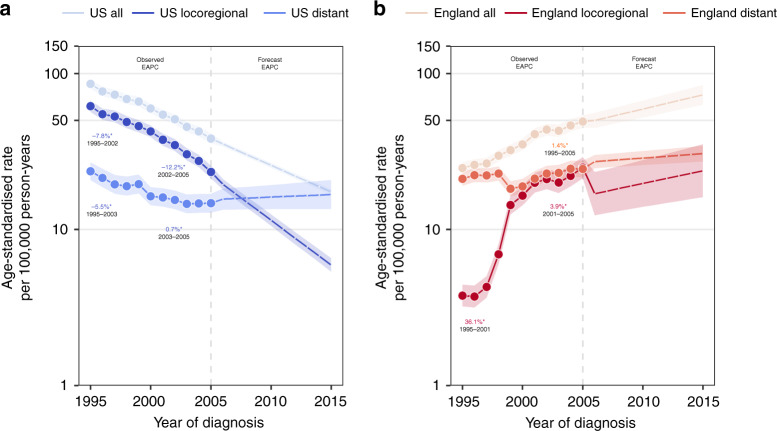
Observed and forecasted fatal prostate cancer incidence in the United States and England, by stage at diagnosis. **a**, **b** Overall, locoregional, and distant stage fatal prostate cancer incidence trends in the United States and England, respectively. Age-standardised rates are presented for men aged 45–84 years. Temporal trends are described using estimates from age–period–cohort, joinpoint, and forecast regression models. Solid and dashed lines represent lines indicate observed and projected trends, respectively. Shaded regions surrounding trend lines indicate 95% confidence intervals. EAPCs with *p* values <0.05 are indicated by asterisk ‘*’. EAPC estimated annual percentage change.

In the US, locoregional prostate cancer that was ultimately fatal decreased between 1995 and 2002 (EAPC: −7.8%) and then more rapidly between 2002 and 2005 (EAPC: −12.2%); our forecast model indicates that this will likely continue for diagnoses through 2015 (Fig. 2a). Meanwhile, distant prostate cancer that was ultimately fatal initially declined (EAPC: −5.5%) before stabilising in 2003 in the US (EAPC: 0.7%), and we project that this trend will remain stable through 2015. Our projections predict that distant stage disease will become the predominant contributor to fatal prostate cancer in the US, beginning with cases diagnosed from 2010 and onward; this observation mirrors overall prostate cancer incidence trends (Supplementary Fig. 1c) in which distant stage has recently increased (EAPC: 4.3% between 2011 and 2015). In England, distant stage disease was the predominant contributor to fatal prostate cancer burden in nearly all periods observed and forecast (Fig. 2b).

**Fig. 3:**
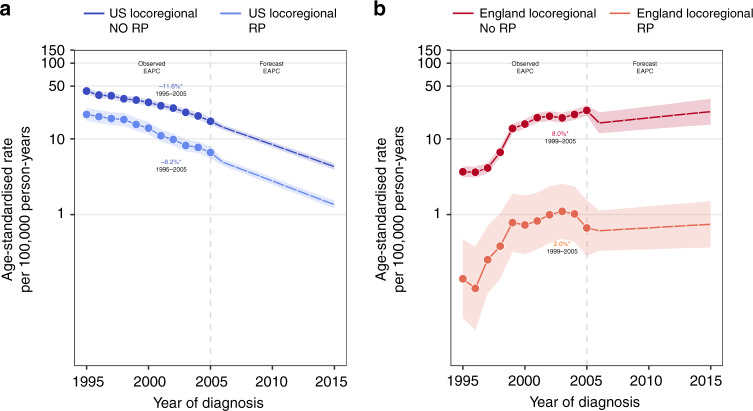
Observed and forecasted fatal prostate cancer incidence in the United States and England, by stage at diagnosis and receipt of radical prostatectomy (RP). **a**, **b** Locoregional and distant stage fatal prostate cancer incidence trends by receipt of RP in the United States and England, respectively. Age-standardised rates are presented for men aged 45–84 years. Temporal trends are described using estimates from age–period–cohort, joinpoint, and forecast regression models. Solid and dashed lines represent lines indicate observed and projected trends, respectively. Shaded regions surrounding trend lines indicate 95% confidence intervals. EAPCs with *p* values <0.05 are indicated by asterisk ‘*’. EAPC estimated annual percentage change.

The overall proportion of men receiving RP in the US remained steady across the study period (~33%), whereas uptake of RP in England gradually increased from 4% in 1995 to 14% in 2015. In the US, 10% of men with fatal prostate cancer had received RP, compared with only 1% of similar men in the English population; in both countries, a higher proportion of men with non-fatal prostate cancer had received RP (US: 35% and England: 9%; data not shown). Fatal prostate cancer incidence rates declined among US men diagnosed with locoregional disease irrespective of RP treatment, with projected declines continuing through 2015 (Fig. 3a). The fatal prostate cancer incidence rate in England remained remarkably low among men diagnosed with locoregional disease and treated by RP, ranging between 3.0 per 100,000 and 4.6 per 100,000 across the study period (EAPC: 2.0%); by contrast, the rate among men who did not receive RP increased at a substantial 8.0% per year between 1999 and 2005.

### Incidence of fatal prostate cancer by country and race

**Fig. 4:**
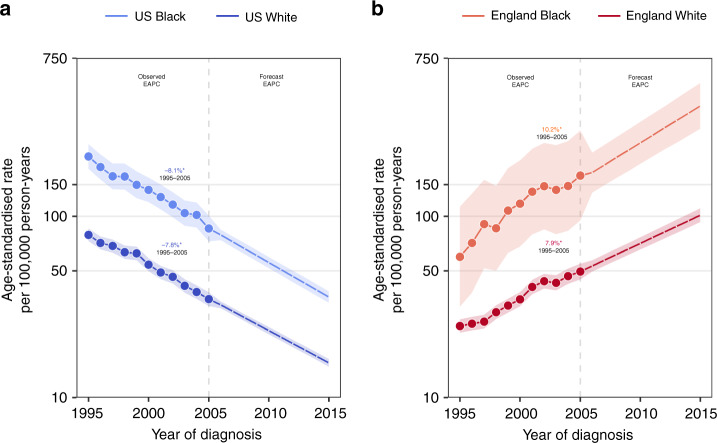
Observed and forecasted fatal prostate cancer incidence in the United States and England by race. **a**, **b** Fatal prostate cancer incidence trends by black or white race in the United States and England, respectively. Age-standardised rates are presented for men aged 45–84 years. Temporal trends are described using estimates from age–period–cohort, joinpoint, and forecast regression models. Solid and dashed lines represent lines indicate observed and projected trends, respectively. Shaded regions surrounding trend lines indicate 95% confidence intervals. EAPCs with *p* values <0.05 are indicated by asterisk ‘*’. EAPC estimated annual percentage change.

Declines in the age-adjusted incidence rates of fatal prostate cancer were similar among black and white men in the US (EAPC: −8.1% vs. −7.8%, Wald’s *Χ*^2^ = 0.4, df = 1, *p* = 0.5; Fig. 4). We observed a similar pattern when comparing black men in England to white men in England (EAPC: 10.2% vs. 7.9%, Wald’s *Χ*^2^ = 2.3, df = 1, *p* = 0.1). Notably, although temporal trends of fatal prostate cancer incidence did not differ between black and white men in either country, the absolute rate of disease remained 2–3 times higher in black men relative to white men in both the US and England. Cross-sectional age-specific incidence rates in the year 2000 were higher for black men in both countries, particularly among younger men aged 45–49 years. For this age group, the black-to-white incidence rate ratio achieved a maximum of 10:1 in the US and a maximum of 5:1 in England (data not shown).

**Fig. 5:**
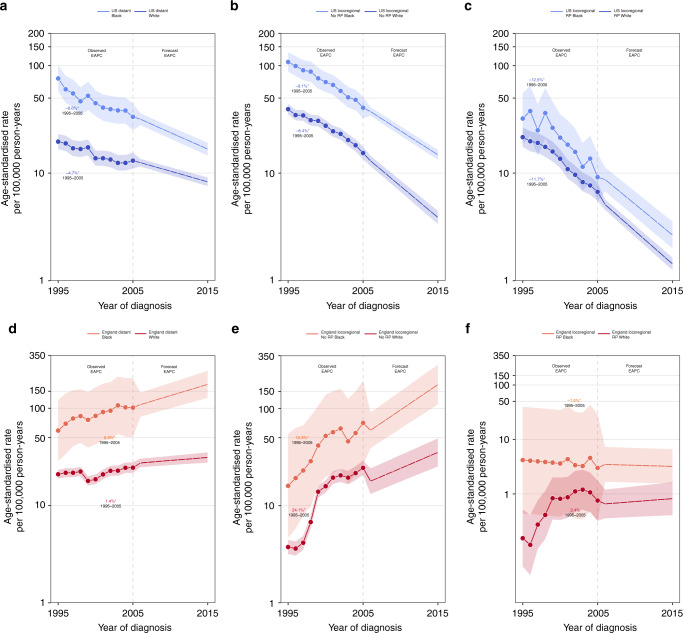
Observed and forecasted fatal prostate cancer incidence in the United States and England by race, stage, and receipt of radical prostatectomy (RP). **a**–**c** Fatal prostate cancer incidence trends in the United States stratified by race for distant stage, locoregional stage without receipt of RP, and locoregional stage with receipt of RP. **d**–**f** Fatal prostate cancer incidence trends in England stratified by race for distant stage, locoregional stage without receipt of RP, and locoregional stage with receipt of RP. Age-standardised rates are presented for men aged 45–84 years. Temporal trends are described using estimates from age–period–cohort, joinpoint, and forecast regression models. Solid and dashed lines represent lines indicate observed and projected trends, respectively. Shaded regions surrounding trend lines indicate 95% confidence intervals. EAPCs with *p* values <0.05 are indicated by asterisk ‘*’. EAPC estimated annual percentage change.

Between 1995 and 2015, black US men overall were less likely to receive RP when compared with white US men (black: 27% vs. white: 34%). Over the same time period, black US men were also slightly more likely to be diagnosed with distant stage disease (black: 6% vs. white: 4%). In England, black men had slightly higher receipt of RP (black: 13% vs. white: 10%) and were less likely to be diagnosed with distant stage disease (black: 20% vs. white: 25%) (data not shown). Race-specific fatal prostate cancer incidence trends (observed and forecast) by stage and treatment mimicked that which was observed in the overall analyses where black and white men experienced similar EAPCs within country (Fig. 5). Nevertheless, black men had higher age-standardised fatal prostate cancer incidence rates when compared with white men, except among those diagnosed with locoregional disease who received RP. In this category, black and white men experienced similar incidence rates within country (Fig. 5c, f).

## Discussion

In this study, we observed opposing trends for fatal prostate cancer incidence in the US and England during a period of evolving clinical practices and screening recommendations. The decreasing and increasing incidence rates in the US and England, respectively, did not differ by race; however, black men in both countries had 2–3-times higher absolute rates of fatal disease across the study period, when compared with their white counterparts. Notably, this disparity was attenuated among men diagnosed with locoregional disease who received an RP. In the US, fatal prostate cancer incidence trends were primarily driven by a decreased risk of death from prostate cancer following diagnosis of locoregional disease, while in England the increased risk of prostate cancer death over time was driven by a mixture of locoregional and distant diseases.

During the early-to-mid 1990s, prostate cancer mortality declined in the US and UK following the advent of PSA testing and RP as a curative measure for localised disease.^5,6,10,15^ After an initial spike, the US prostate cancer incidence rate decreased concurrently with declining mortality as the UK incidence rate continued to rise. Sharp increases in prostate cancer incidence have largely been attributed to the uptake of PSA testing, accounting for a 2–6% higher number of cases than expected in England and as much as a 15–37% higher number of cases than expected in the US.^10,31^ At present, rates of PSA screening in the UK remain low, although increasing incidence in the country—particularly among men aged <70 years—may be indicative of growing PSA test use.^32^ In the US, several entities have issued screening recommendations, most notably among them the United States Preventive Services Task Force whose recommendations have remained conservative given concerns of overdiagnosis and overtreatment. Results from the UK-based Cluster Randomized Trial of PSA Testing for Prostate Cancer (CAP) led an expert panel to issue a guideline recommendation against systematic screening, which also emphasised the need for shared decision-making between patients and clinicians.^7,33^

The higher incidence rate for locoregional disease among US men is likely attributable to greater PSA test use in the US compared with the UK.^13,14,34,35^ PSA testing conflates traditional measures of disease burden by increasing the detection of indolent tumours that may have otherwise gone unobserved. Studies that have reported changes in incidence or improvements in prostate cancer survival often do not discriminate between indolent and non-indolent cancers, thereby affecting and obscuring true changes in disease metrics.^36^ Thus, by describing fatal prostate cancer incidence in the US and England, our study provides a unique evaluation of temporal trends for a clinically relevant subset of the disease in a comparison of two countries with substantially different PSA test use histories. Further, our age-specific analyses demonstrate that the direction of within-country trends do not differ by age; in both countries, however, fatal prostate cancer incidence rates decrease (US) or increase (England) to a greater degree among younger men. This finding is particularly important for the English population, where the rate of RP uptake remains stable despite increasing incidence rates for overall and fatal disease. The use of RP or other putative curative treatments may be important for addressing the higher incidence rate of fatal disease among young English men.

As previously reported, fatal prostate cancer incidence has been decreasing among US men since the early 1990s, and by 2002, the incidence rate had declined by approximately 50% of its 1975 estimate.^1^ In the present study, we have demonstrated the continued decrease in US rates through the year 2005 and we project that this trend will continue through 2015. Notably, our forecast models predict that distant stage disease is likely to become the predominant contributor to fatal prostate cancer incidence in the US for the year 2010 and forward. As prostate cancer incidence in the US continues to decline, the overall stage distribution has shifted to an increasing proportion of distant diagnoses following declines in PSA test use.^37^ Additional years of data are needed to determine whether the US fatal prostate cancer incidence rate will continue to decline and stabilise. By contrast, the fatal prostate cancer incidence rate has increased in England, doubling between 1995 and 2005. Given that prostate cancer incidence is projected to increase in the UK through the year 2035,^17^ this observation serves as an impetus to explore individual- and system-level factors that may be linked to risk of fatal disease.

Black men in both the US and UK experience higher total prostate cancer incidence rates compared with white men,^20^ and as evidenced by the present study, this differential persists for incidence of fatal disease. We currently have a limited understanding of prostate cancer aetiology, including to what extent social or biological factors may contribute to higher incidence in black men compared with men of other ancestries. Notably, however, findings from our study support recent observations that equal treatment for equal disease may lead to equal outcomes for black and white prostate cancer patients,^38,39^ as there was no apparent race disparity in fatal prostate cancer incidence among men diagnosed with locoregional disease who received an RP. Thus ensuring access to RP is likely to be an effective strategy in reducing fatal prostate cancer incidence among black men. And though outside the scope of our present analysis, it will be important for future studies to examine the influence of other treatment regimens on fatal prostate cancer incidence by geography, treatment, and race/ethnicity.

Our study benefits from the use of population-based cancer registry data with adequate follow-up to ascertain vital status and cause of death during the 10-year period following cancer diagnosis. In addition, we described observed trends for the incidence of locoregional and distant stage trends through 2015 to bolster our interpretation of projected trends for fatal prostate cancer incidence. It is important to note that our projections of locoregional and distant disease may differ from future trends as contemporary advances in imaging increase our ability to detect metastases. It is also important to note that the population sample provided through SEER may not represent all US men. Further, the nature of our ecological study design did not allow us to describe associations between individual-level treatment patterns or timing of PSA use; however, our analytical approach with age–period–cohort models allowed us to estimate period effects that coincide with temporal clinical practices for screening and surgical treatment. The high proportion of missing data in the English data set should also be considered when interpreting our findings.

By examining the incidence of fatal prostate cancer in the US and England, our study offers insights on trends for a clinically relevant subset of the disease. Our study demonstrates a dramatic increase in fatal prostate cancer incidence among English men that warrants public health concern, particularly among young and black men. The persistent black–white race disparity in the US and England also warrants further consideration in order to alleviate excess burden of disease in the most vulnerable populations.

## Supplementary information

Supplementary Material

## Data Availability

The cancer registry data from the United States is available upon request to the Surveillance, Epidemiology, and End Results Program and can be reached at seertrack@imsweb.com. Inquiries for England’s cancer registration data can be made to Public Health England’s Office for Data Release at ODR@phe.gov.uk.

## References

[1] Kelly SP, Rosenberg PS, Anderson WF, Andreotti G, Younes N, Cleary SD (2017). Trends in the incidence of fatal prostate cancer in the United States by race. Eur. Urol..

[2] Epstein MM, Edgren G, Rider JR, Mucci LA, Adami HO (2012). Temporal trends in cause of death among Swedish and US men with prostate cancer. J. Natl Cancer Inst..

[3] Andriole GL (1992). Serum prostate-specific antigen: the most useful tumor marker. J. Clin. Oncol..

[4] Lu-Yao GL, Friedman M, Yao SL (1997). Use of radical prostatectomy among Medicare beneficiaries before and after the introduction of prostate specific antigen testing. J. Urol..

[5] Sheikh K, Bullock C (2002). Rise and fall of radical prostatectomy rates from 1989 to 1996. Urology.

[6] Collin SM, Martin RM, Metcalfe C, Gunnell D, Albertsen PC, Neal D (2008). Prostate-cancer mortality in the USA and UK in 1975-2004: an ecological study. Lancet Oncol..

[7] Martin RM, Donovan JL, Turner EL, Metcalfe C, Young GJ, Walsh EI (2018). Effect of a low-intensity PSA-based screening intervention on prostate cancer mortality: the CAP Randomized clinical trial. JAMA.

[8] Schroder FH, Hugosson J, Roobol MJ, Tammela TL, Ciatto S, Nelen V (2009). Screening and prostate-cancer mortality in a randomized European study. N. Engl. J. Med..

[9] Andriole GL, Crawford ED, Grubb RL, Buys SS, Chia D, Church TR (2009). Mortality results from a randomized prostate-cancer screening trial. N. Engl. J. Med..

[10] Etzioni R, Penson DF, Legler JM, di Tommaso D, Boer R, Gann PH (2002). Overdiagnosis due to prostate-specific antigen screening: lessons from US prostate cancer incidence trends. J. Natl Cancer Inst..

[11] Force USPST, Grossman DC, Curry SJ, Owens DK, Bibbins-Domingo K, Caughey AB (2018). Screening for prostate cancer: US Preventive Services Task Force Recommendation Statement. JAMA.

[12] Patel NH, Bloom J, Hillelsohn J, Fullerton S, Allman D, Matthews G (2018). Prostate cancer screening trends after United States Preventative Services Task Force Guidelines in an underserved population. Health Equity.

[13] Melia J, Moss S, Johns L (2004). Contributors in the participating l. Rates of prostate-specific antigen testing in general practice in England and Wales in asymptomatic and symptomatic patients: a cross-sectional study. BJU Int..

[14] Williams N, Hughes LJ, Turner EL, Donovan JL, Hamdy FC, Neal DE (2011). Prostate-specific antigen testing rates remain low in UK general practice: a cross-sectional study in six English cities. BJU Int..

[15] Melia J, Moss S (2001). Survey of the rate of PSA testing in general practice. Br. J. Cancer.

[16] Hall IJ, Tangka FKL, Sabatino SA, Thompson TD, Graubard BI, Breen N (2018). Patterns and trends in cancer screening in the United States. Prev. Chronic Dis..

[17] Smittenaar CR, Petersen KA, Stewart K, Moitt N (2016). Cancer incidence and mortality projections in the UK until 2035. Br. J. Cancer.

[18] Jack RH, Davies EA, Moller H (2010). Prostate cancer incidence, stage at diagnosis, treatment and survival in ethnic groups in South-East England. BJU Int..

[19] Cook MB, Rosenberg PS, McCarty FA, Wu M, King J, Eheman C (2015). Racial disparities in prostate cancer incidence rates by census division in the United States, 1999-2008. Prostate.

[20] Ben-Shlomo Y, Evans S, Ibrahim F, Patel B, Anson K, Chinegwundoh F (2008). The risk of prostate cancer amongst black men in the United Kingdom: the PROCESS cohort study. Eur. Urol..

[21] National Cancer Institute, DCCPS, Surveillance Research Program. Surveillance, Epidemiology, and End Results (SEER) Program (www.seer.cancer.gov) SEER*Stat Database: Incidence - SEER 18 Regs Research Data, Nov 2017 Sub (1973-2015) <Katrina/Rita Population Adjustment> - Linked To County Attributes - Total U.S., 1969-2016 Counties. https://seer.cancer.gov/data-software/documentation/seerstat/nov2017/ (2018).

[22] National Disease Registration Service. https://www.ndrs.nhs.uk/. (2018). Accessed 6 Dec 2018.

[23] van Buuren S, Groothuis-Oudshoorn K (2011). mice: Multivariate imputation by chained equations in R. J. Stat. Softw..

[24] Rubin DB (1987). Multiple Imputation for Nonresponse in Surveys.

[25] Ahmad, O. B., Boschi-Pinto, C., Lopez, A. D., Murray, C. J. L., Lozano, R. & Inoue, M. *Age Standardization of Rates: A New WHO Standard* (World Health Organization, Geneva, 2001).

[26] Kim HJ, Fay MP, Feuer EJ, Midthune DN (2000). Permutation tests for joinpoint regression with applications to cancer rates. Stat. Med..

[27] Rosenberg PS, Anderson WF (2011). Age-period-cohort models in cancer surveillance research: ready for prime time?. Cancer Epidemiol. Biomark. Prev..

[28] Holford TR (1983). The estimation of age, period and cohort effects for vital rates. Biometrics.

[29] Rosenberg PS, Anderson WF (2010). Proportional hazards models and age-period-cohort analysis of cancer rates. Stat. Med..

[30] Rosenberg, P. S., Barker, K. A. & Anderson, W. F. Estrogen receptor status and the future burden of invasive and in situ breast cancers in the United States. *J. Natl Cancer Inst*. **107**, djv159 (2015) 10.1093/jnci/djv159 PMC4836802 26063794

[31] Pashayan N, Powles J, Brown C, Duffy SW (2006). Incidence trends of prostate cancer in East Anglia, before and during the era of PSA diagnostic testing. Br. J. Cancer.

[32] Moller H, Fairley L, Coupland V, Okello C, Green M, Forman D (2007). The future burden of cancer in England: incidence and numbers of new patients in 2020. Br. J. Cancer.

[33] Tikkinen KAO, Dahm P, Lytvyn L, Heen AF, Vernooij RWM, Siemieniuk RAC (2018). Prostate cancer screening with prostate-specific antigen (PSA) test: a clinical practice guideline. BMJ.

[34] Wallner L, Frencher S, Hsu JW, Loo R, Huang J, Nichol M (2012). Prostate cancer screening trends in a large, integrated health care system. Perm. J..

[35] Sirovich BE, Schwartz LM, Woloshin S (2003). Screening men for prostate and colorectal cancer in the United States: does practice reflect the evidence?. JAMA.

[36] Etzioni R, Gulati R, Mallinger L, Mandelblatt J (2013). Influence of study features and methods on overdiagnosis estimates in breast and prostate cancer screening. Ann. Intern. Med..

[37] Fleshner K, Carlsson SV, Roobol MJ (2017). The effect of the USPSTF PSA screening recommendation on prostate cancer incidence patterns in the USA. Nat. Rev. Urol..

[38] Dess RT, Hartman HE, Mahal BA, Soni PD, Jackson WC, Cooperberg MR (2019). Association of black race with prostate cancer-specific and other-cause mortality. JAMA Oncol..

[39] Paller CJ, Wang L, Brawley OW (2019). Racial inequality in prostate cancer outcomes-socioeconomics, not biology. JAMA Oncol..

