# Supplementary material for: Fatal prostate cancer incidence trends in the United States and England by race, stage, and treatment
Source: Br J Cancer. 2020 May 20;123(3):487–94. doi: 10.1038/s41416-020-0859-x (PMC7403310; doi:10.1038/s41416-020-0859-x)
Supplement: Supplementary file 1 — Supplementary Material [file 41416_2020_859_MOESM1_ESM.docx]

**LEGENDS**

**Supplemental Figure 1**

Age-standardized overall prostate cancer incidence rates in the United States and England, by stage at diagnosis for men aged 45 to 84 years (1995-2015). Incidence rates are presented on a log scale.

**Supplemental Figure 1**


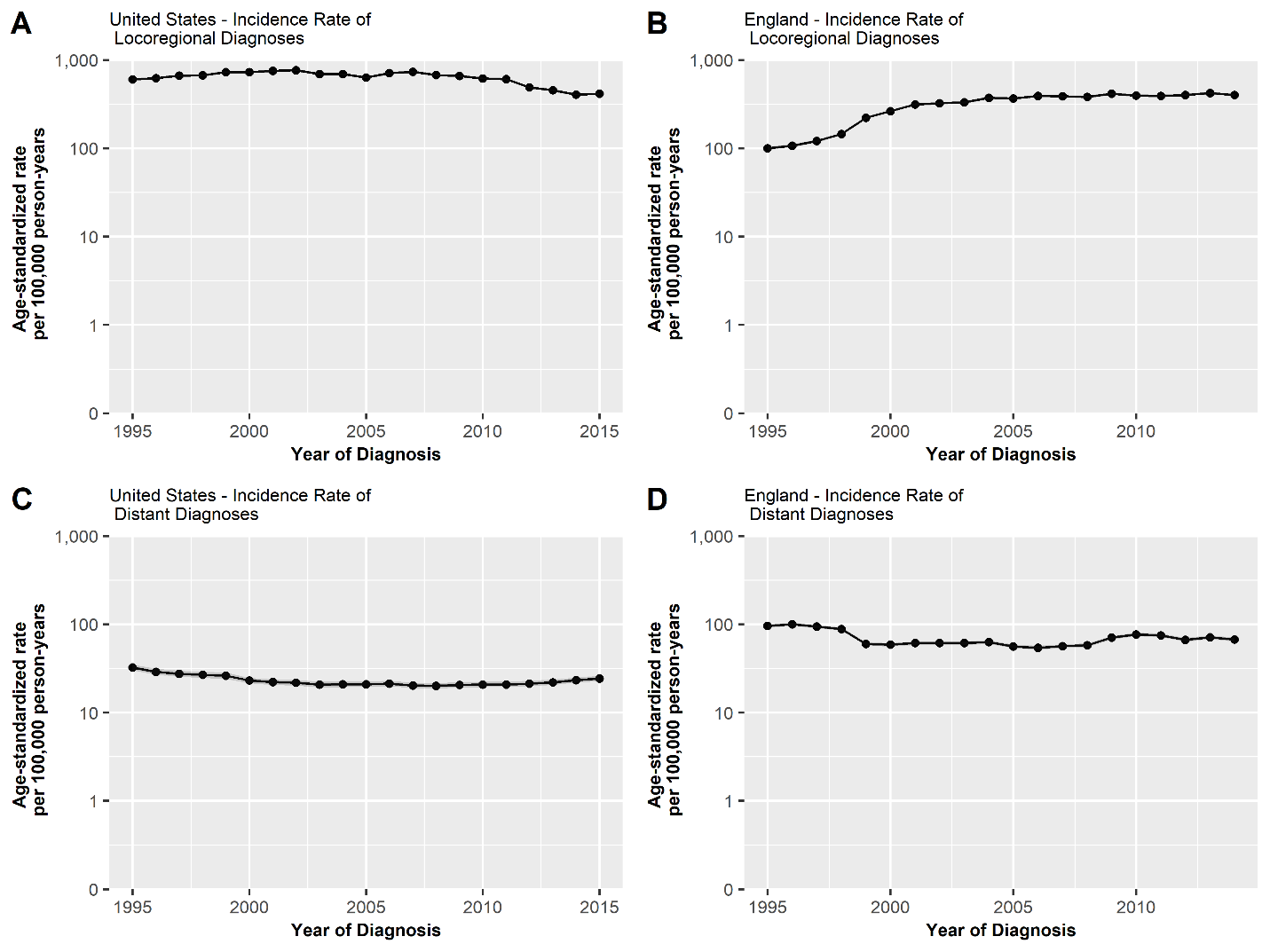


Supplemental Table 1. Beta coefficients for the associations between race, treatment, stage, grade, and prostate cancer-specific death across multiple imputation models in the US dataset.

| Model Estimate | Pooled | M1 | M2 | M3 | M4 | M5 | M6 | M7 | M8 | M9 | M10 |
| --- | --- | --- | --- | --- | --- | --- | --- | --- | --- | --- | --- |
| (Intercept) | -0.79 | -0.81 | -0.80 | -0.78 | -0.80 | -0.80 | -0.80 | -0.78 | -0.80 | -0.79 | -0.79 |
| Race = Other | -0.38 | -0.38 | -0.38 | -0.38 | -0.38 | -0.38 | -0.38 | -0.38 | -0.38 | -0.38 | -0.38 |
| Race = White | -0.04 | -0.04 | -0.45 | -0.04 | -0.04 | -0.05 | -0.04 | -0.04 | -0.46 | -0.46 | -0.04 |
| Surgery = Other | 1.05 | 1.06 | 1.05 | 1.06 | 1.06 | 1.05 | 1.05 | 1.05 | 1.05 | 1.05 | 1.05 |
| Surgery = RP | -1.34 | -1.30 | -1.30 | -1.30 | -1.30 | -1.30 | -1.30 | -1.30 | -1.30 | -1.30 | -1.30 |
| Stage = Locoregional | -2.99 | -2.99 | -2.99 | -2.99 | -2.98 | -2.98 | -2.99 | -2.99 | -2.98 | -2.98 | -2.98 |
| Grade = II | 1.05 | 1.06 | 1.05 | 1.03 | 1.05 | 1.05 | 1.05 | 1.03 | 1.05 | 1.04 | 1.04 |
| Grade = III | 1.96 | 1.97 | 1.97 | 1.94 | 1.97 | 1.96 | 1.97 | 1.94 | 1.96 | 1.96 | 1.96 |
| Grade = IV | 3.24 | 3.30 | 3.30 | 3.20 | 3.30 | 3.20 | 3.20 | 3.30 | 3.20 | 3.20 | 3.20 |

*Note.* Abbreviations: M1-M10 – Multiple Imputations; RP – Radical Prostatectomy. Beta coefficients were obtained from multinomial models examining the associations between imputed variables and cause of death (prostate cancer, other, alive). Referent groups were as follows: Race (‘Black’), Surgery (‘None’), Stage (‘Distant’), and Grade (‘I’). Coefficients are presented for “prostate cancer-specific death” only.

Supplemental Table 2. Beta coefficients for the associations between race, stage, grade, and prostate cancer-specific death across multiple imputation models in the English dataset.

| Model Estimate | Pooled | M1 | M2 | M3 | M4 | M5 | M6 | M7 | M8 | M9 | M10 |
| --- | --- | --- | --- | --- | --- | --- | --- | --- | --- | --- | --- |
| (Intercept) | -3.25 | -3.33 | -3.01 | -3.31 | -3.06 | -3.30 | -3.09 | -3.17 | -3.04 | -3.97 | -3.22 |
| Race = Black | -0.79 | -0.80 | -0.78 | -0.74 | -0.80 | -0.86 | -0.79 | -0.75 | -0.79 | -0.81 | -0.79 |
| Race = Other | -0.79 | -0.77 | -0.57 | -0.77 | -0.90 | -0.75 | -0.85 | -0.76 | -0.80 | -0.84 | -0.93 |
| Stage = 2 | 2.06 | 2.11 | 2.02 | 2.09 | 1.95 | 2.08 | 1.96 | 2.09 | 2.12 | 2.10 | 2.08 |
| Stage = 3 | 1.90 | 1.95 | 1.86 | 1.92 | 1.81 | 1.96 | 1.81 | 1.93 | 1.95 | 1.95 | 1.91 |
| Stage = 4 | 4.46 | 4.52 | 4.42 | 4.45 | 4.36 | 4.50 | 4.41 | 4.47 | 4.50 | 4.50 | 4.47 |
| Combined Grade = 3 | -0.56 | -0.44 | -0.67 | -0.63 | -0.98 | -0.52 | -0.53 | -0.58 | -0.81 | 0.27 | -0.73 |
| Combined Grade = 4 | -0.42 | -0.36 | -0.62 | -0.46 | -0.56 | -0.35 | -0.46 | -0.52 | -0.71 | 0.35 | -0.47 |
| Combined Grade = 5 | -0.52 | -0.48 | -0.69 | -0.58 | -0.61 | -0.55 | -0.35 | -0.69 | -0.81 | 0.14 | -0.61 |
| Combined Grade = 6 | -1.59 | -1.57 | -1.79 | -1.57 | -1.68 | -1.57 | -1.69 | -1.68 | -1.85 | -0.86 | -1.64 |
| Combined Grade = 7 | -1.42 | -1.42 | -1.64 | -1.38 | -1.49 | -1.42 | -1.49 | -1.52 | -1.70 | -0.74 | -1.44 |
| Combined Grade = 8 | -0.73 | -0.65 | -0.89 | -0.60 | -0.84 | -0.71 | -0.85 | -0.83 | -0.96 | -0.12 | -0.87 |
| Combined Grade = 9 | -0.94 | -0.91 | -1.18 | -0.88 | -1.04 | -0.93 | -1.08 | -1.04 | -1.21 | -0.22 | -0.88 |
| Combined Grade = 10 | -0.09 | -0.15 | -0.30 | -0.07 | -0.18 | -0.15 | -0.28 | -0.14 | -0.41 | 0.88 | -0.06 |

*Note.* Abbreviation: M1-M10 – Multiple Imputations. Beta coefficients were obtained from multinomial models examining the associations between imputed variables and cause of death (prostate cancer, other, alive). Referent groups were as follows: Race (‘White’), Stage (‘1’), and Combined Grade (‘2’). Coefficients are presented for “prostate cancer-specific death” only.
